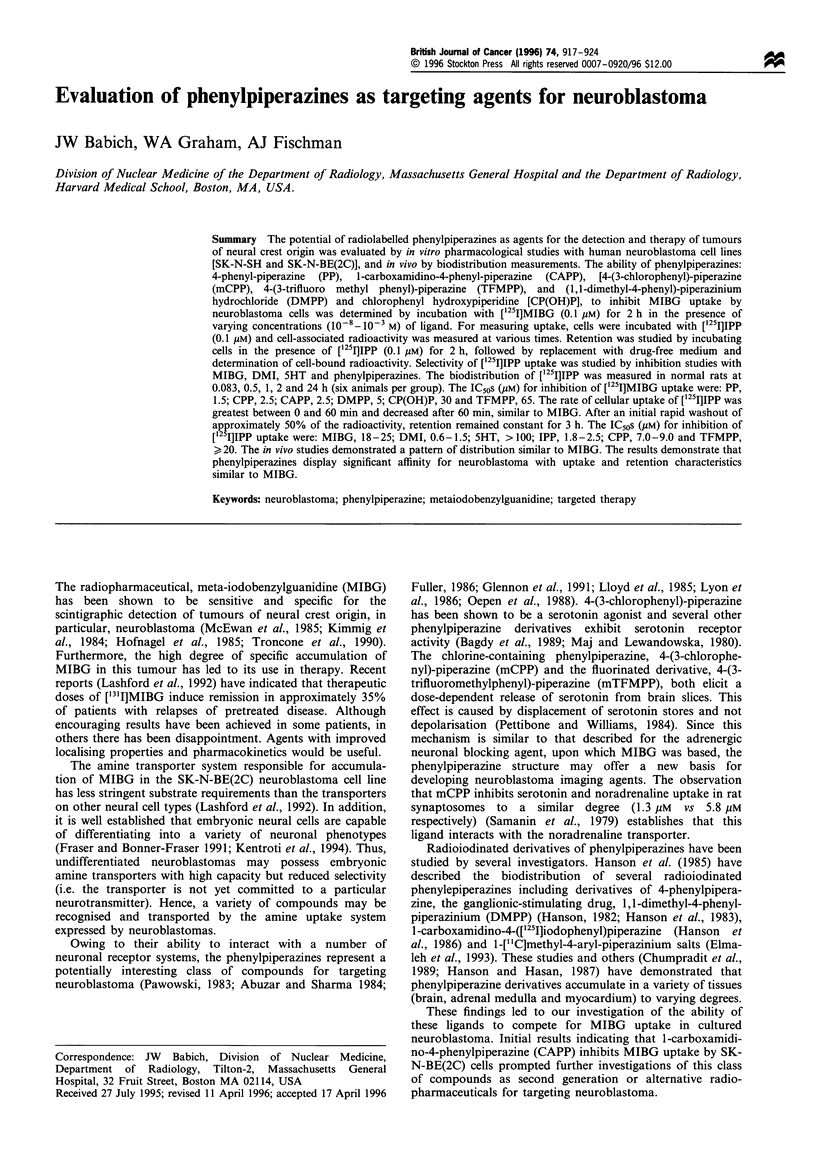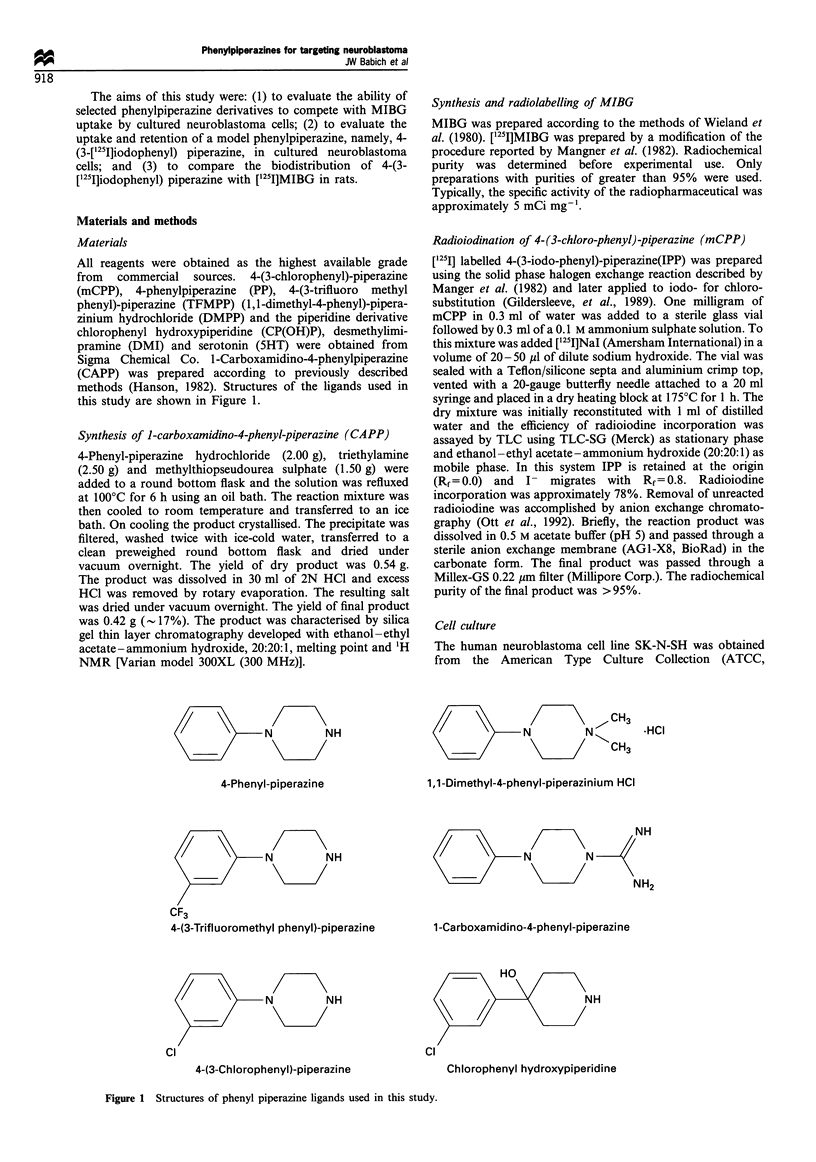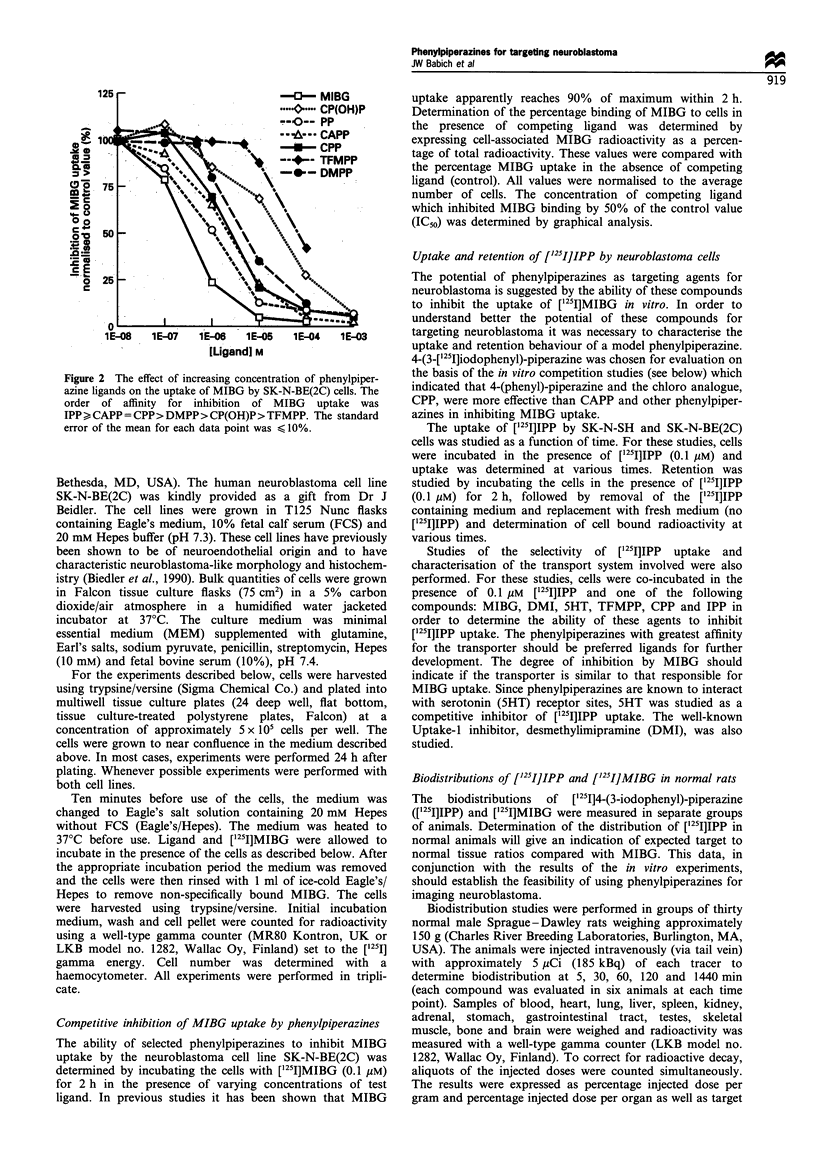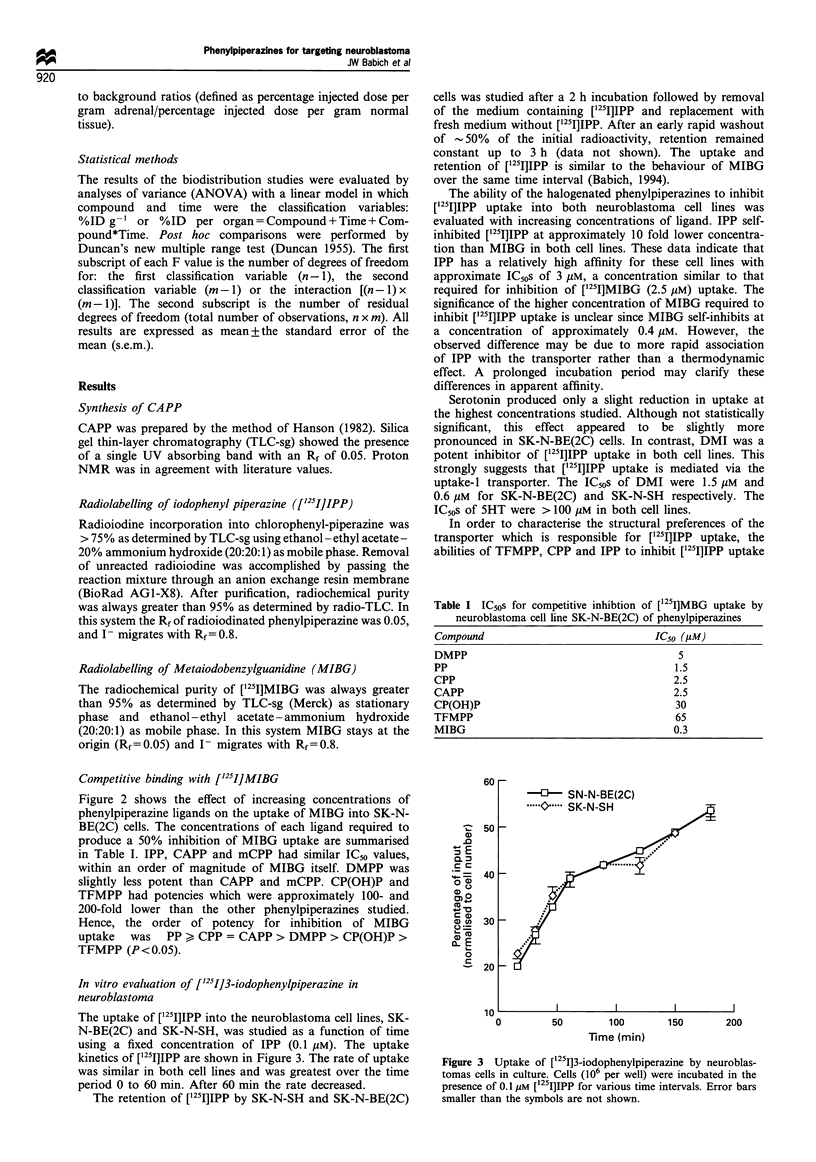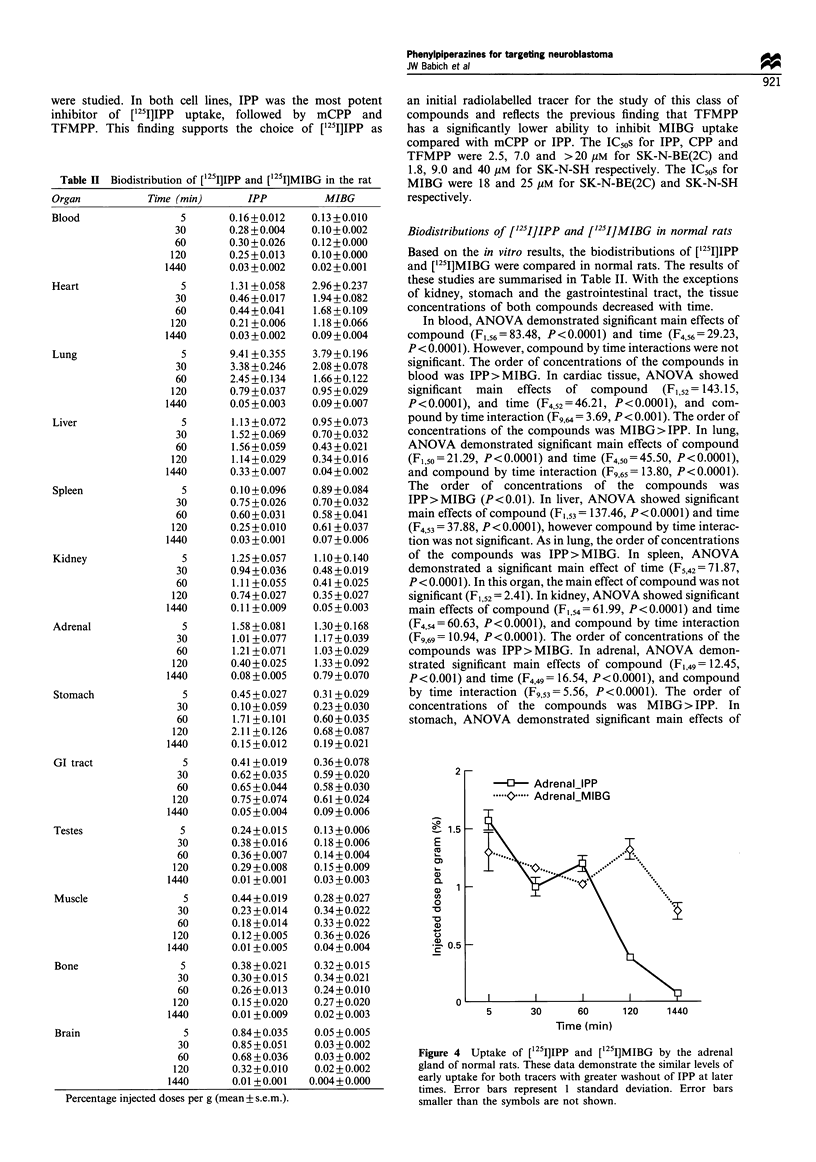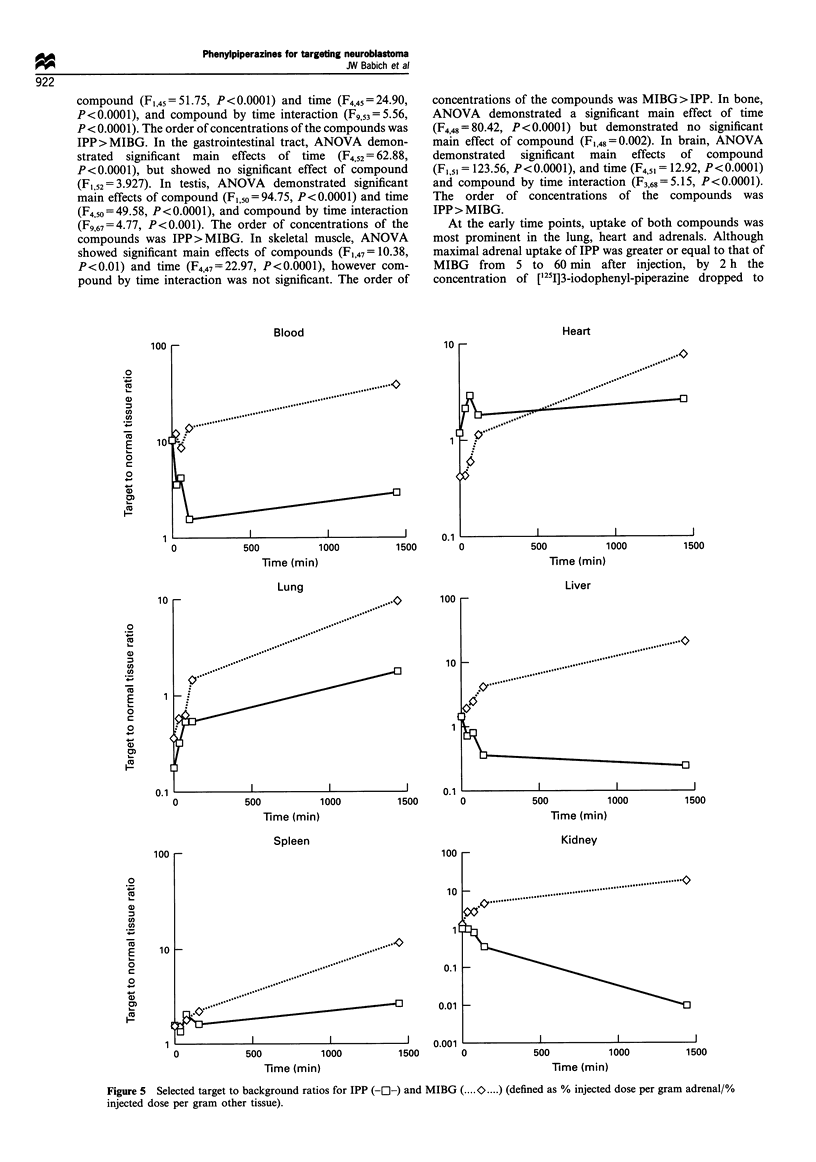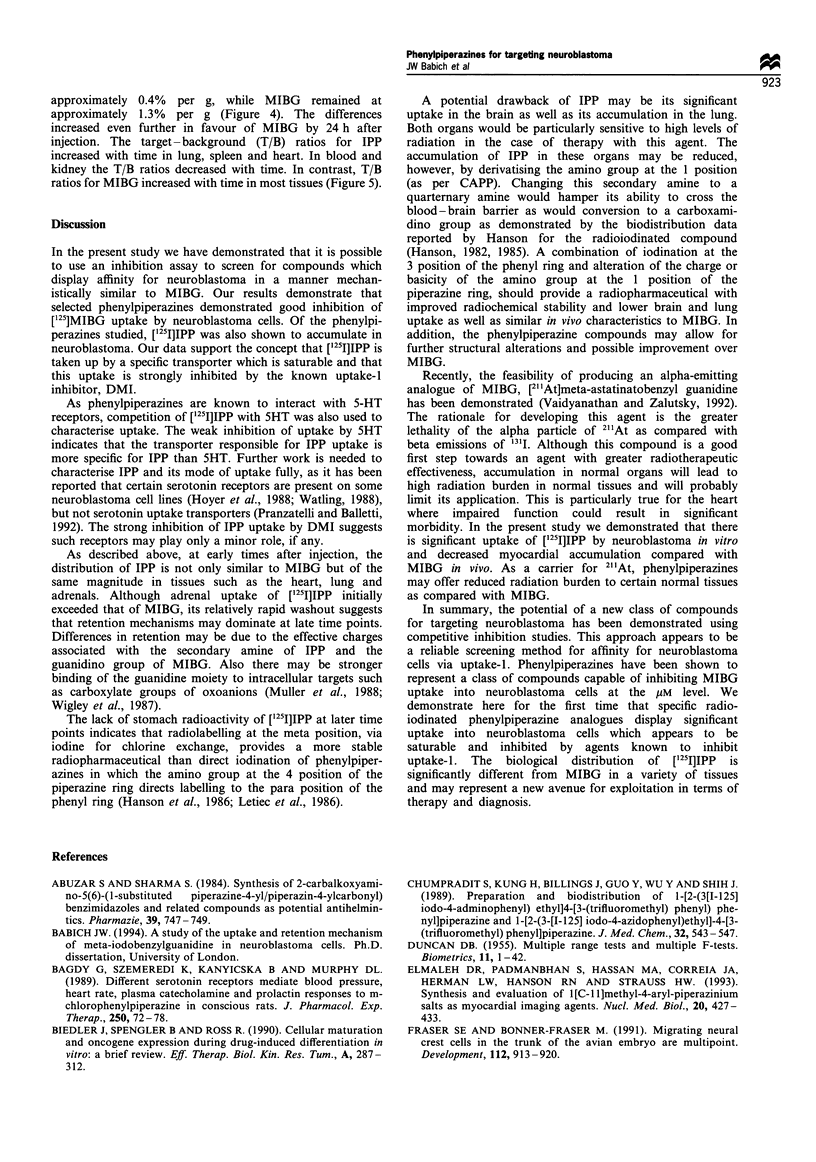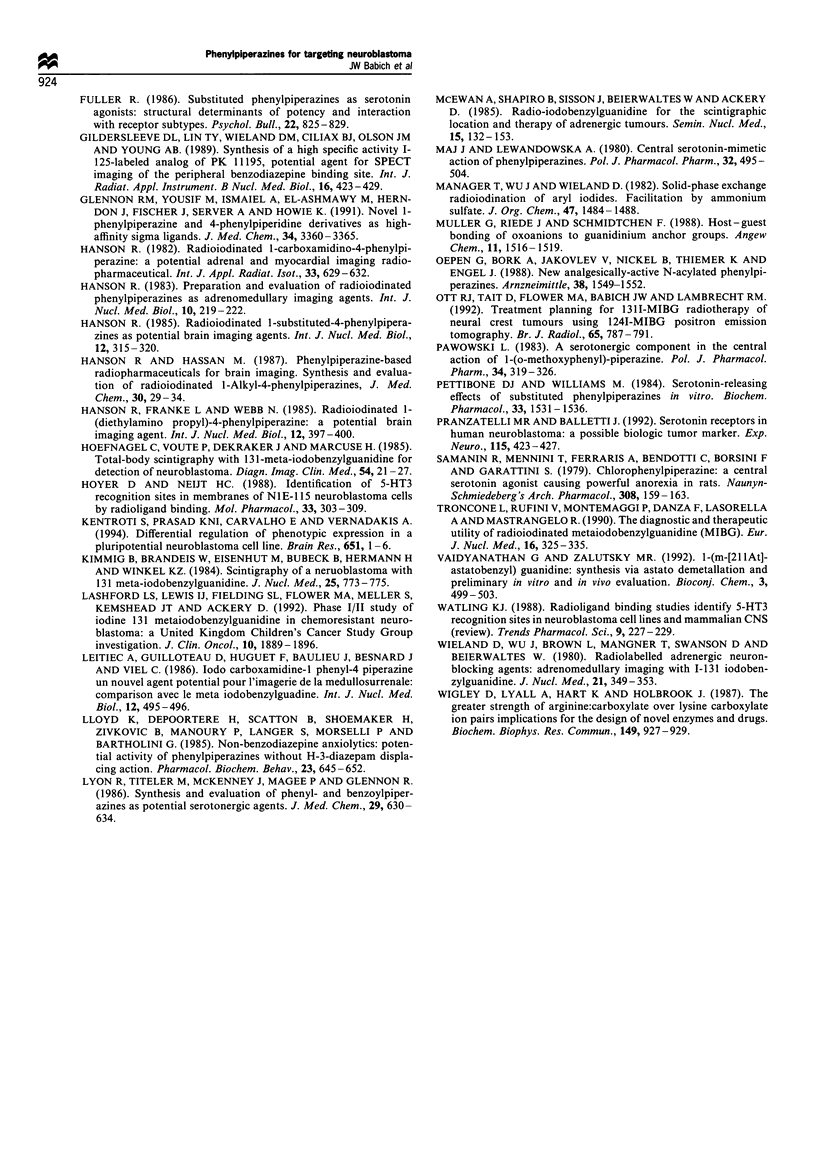# Evaluation of phenylpiperazines as targeting agents for neuroblastoma.

**DOI:** 10.1038/bjc.1996.457

**Published:** 1996-09

**Authors:** J. W. Babich, W. A. Graham, A. J. Fischman

**Affiliations:** Department of Radiology, Massachusetts General Hospital, Boston, USA.

## Abstract

The potential of radiolabelled phenylpiperazines as agents for the detection and therapy of tumours of neural crest origin was evaluated by in vitro pharmacological studies with human neuroblastoma cell lines [SK-N-SH and SK-N-BE(2C)], and in vivo by biodistribution measurements. The ability of phenylpiperazines: 4-phenyl-piperazine (PP), 1-carboxamidino-4-phenyl-piperazine (CAPP), [4-(3-chlorophenyl)-piperazine (mCPP), 4-(3-trifluoro methyl phenyl)-piperazine (TFMPP), and (1,1-dimethyl-4-phenyl)-piperazinium hydrochloride (DMPP) and chlorophenyl hydroxypiperidine [CP(OH)P], to inhibit MIBG uptake by neuroblastoma cells was determined by incubation with [125I]MIBG (0.1 microM) for 2 h in the presence of varying concentrations (10(-8)-10(-3) M) of ligand. For measuring uptake, cells were incubated with [125I]IPP (0.1 microM) and cell-associated radioactivity was measured at various times. Retention was studied by incubating cells in the presence of [125I]IPP (0.1 microM) for 2 h, followed by replacement with drug-free medium and determination of cell-bound radioactivity. Selectivity of [125I]IPP uptake was studied by inhibition studies with MIBG, DMI, 5HT and phenylpiperazines. The biodistribution of [125I]IPP was measured in normal rats at 0.083, 0.5, 1, 2 and 24 h (six animals per group). The IC50S (microM) for inhibition of [125I]MIBG uptake were: PP, 1.5; CPP, 2.5; CAPP, 2.5; DMPP, 5; CP(OH)P, 30 and TFMPP, 65. The rate of cellular uptake of [125I]IPP was greatest between 0 and 60 min and decreased after 60 min, similar to MIBG. After an initial rapid washout of approximately 50% of the radioactivity, retention remained constant for 3 h. The IC50S (microM) for inhibition of [125I]IPP uptake were: MIBG, 18-25; DMI, 0.6-1.5; 5HT, > 100; IPP, 1.8-2.5; CPP, 7.0-9.0 and TFMPP, > or = 20. The in vivo studies demonstrated a pattern of distribution similar to MIBG. The results demonstrate that phenylpiperazines display significant affinity for neuroblastoma with uptake and retention characteristics similar to MIBG.


British Journal of Cancer (1996) 74, 917–924
© 1996 Stockton Press All rights reserved 0007–0920/96 $12.00

### Evaluation of phenylpiperazines as targeting agents for neuroblastoma

JW Babich, WA Graham, AJ Fischman

*Division of Nuclear Medicine of the Department of Radiology, Massachusetts General Hospital and the Department of Radiology, Harvard Medical School, Boston, MA, USA.*

**Summary** The potential of radiolabelled phenylpiperazines as agents for the detection and therapy of tumours of neural crest origin was evaluated by *in vitro* pharmacological studies with human neuroblastoma cell lines [SK-N-SH and SK-N-BE(2C)], and *in vivo* by biodistribution measurements. The ability of phenylpiperazines: 4-phenyl-piperazine (PP), 1-carboxamidino-4-phenyl-piperazine (CAPP), [4-(3-chlorophenyl)-piperazine (mCPP), 4-(3-trifluoro methyl phenyl)-piperazine (TFMPP), and (1,1-dimethyl-4-phenyl)-piperazinium hydrochloride (DMPP) and chlorophenyl hydroxypiperidine [CP(OH)P], to inhibit MIBG uptake by neuroblastoma cells was determined by incubation with [$^{125}$I]MIBG (0.1 $\mu$M) for 2 h in the presence of varying concentrations ($10^{-8}-10^{-3}$ M) of ligand. For measuring uptake, cells were incubated with [$^{125}$I]IPP (0.1 $\mu$M) and cell-associated radioactivity was measured at various times. Retention was studied by incubating cells in the presence of [$^{125}$I]IPP (0.1 $\mu$M) for 2 h, followed by replacement with drug-free medium and determination of cell-bound radioactivity. Selectivity of [$^{125}$I]IPP uptake was studied by inhibition studies with MIBG, DMI, 5HT and phenylpiperazines. The biodistribution of [$^{125}$I]IPP was measured in normal rats at 0.083, 0.5, 1, 2 and 24 h (six animals per group). The $IC_{50}$s ($\mu$M) for inhibition of [$^{125}$I]MIBG uptake were: PP, 1.5; CPP, 2.5; CAPP, 2.5; DMPP, 5; CP(OH)P, 30 and TFMPP, 65. The rate of cellular uptake of [$^{125}$I]IPP was greatest between 0 and 60 min and decreased after 60 min, similar to MIBG. After an initial rapid washout of approximately 50% of the radioactivity, retention remained constant for 3 h. The $IC_{50}$s ($\mu$M) for inhibition of [$^{125}$I]IPP uptake were: MIBG, 18–25; DMI, 0.6–1.5; 5HT, >100; IPP, 1.8–2.5; CPP, 7.0–9.0 and TFMPP, $\geqslant$20. The *in vivo* studies demonstrated a pattern of distribution similar to MIBG. The results demonstrate that phenylpiperazines display significant affinity for neuroblastoma with uptake and retention characteristics similar to MIBG.

**Keywords:** neuroblastoma; phenylpiperazine; metaiodobenzylguanidine; targeted therapy

The radiopharmaceutical, meta-iodobenzylguanidine (MIBG) has been shown to be sensitive and specific for the scintigraphic detection of tumours of neural crest origin, in particular, neuroblastoma (McEwan *et al.*, 1985; Kimmig *et al.*, 1984; Hofnagel *et al.*, 1985; Troncone *et al.*, 1990). Furthermore, the high degree of specific accumulation of MIBG in this tumour has led to its use in therapy. Recent reports (Lashford *et al.*, 1992) have indicated that therapeutic doses of [$^{131}$I]MIBG induce remission in approximately 35% of patients with relapses of pretreated disease. Although encouraging results have been achieved in some patients, in others there has been disappointment. Agents with improved localising properties and pharmacokinetics would be useful.

The amine transporter system responsible for accumulation of MIBG in the SK-N-BE(2C) neuroblastoma cell line has less stringent substrate requirements than the transporters on other neural cell types (Lashford *et al.*, 1992). In addition, it is well established that embryonic neural cells are capable of differentiating into a variety of neuronal phenotypes (Fraser and Bonner-Fraser 1991; Kentroti *et al.*, 1994). Thus, undifferentiated neuroblastomas may possess embryonic amine transporters with high capacity but reduced selectivity (i.e. the transporter is not yet committed to a particular neurotransmitter). Hence, a variety of compounds may be recognised and transported by the amine uptake system expressed by neuroblastomas.

Owing to their ability to interact with a number of neuronal receptor systems, the phenylpiperazines represent a potentially interesting class of compounds for targeting neuroblastoma (Pawowski, 1983; Abuzar and Sharma 1984; Fuller, 1986; Glennon *et al.*, 1991; Lloyd *et al.*, 1985; Lyon *et al.*, 1986; Oepen *et al.*, 1988). 4-(3-chlorophenyl)-piperazine has been shown to be a serotonin agonist and several other phenylpiperazine derivatives exhibit serotonin receptor activity (Bagdy *et al.*, 1989; Maj and Lewandowska, 1980). The chlorine-containing phenylpiperazine, 4-(3-chlorophenyl)-piperazine (mCPP) and the fluorinated derivative, 4-(3-trifluoromethylphenyl)-piperazine (mTFMPP), both elicit a dose-dependent release of serotonin from brain slices. This effect is caused by displacement of serotonin stores and not depolarisation (Pettibone and Williams, 1984). Since this mechanism is similar to that described for the adrenergic neuronal blocking agent, upon which MIBG was based, the phenylpiperazine structure may offer a new basis for developing neuroblastoma imaging agents. The observation that mCPP inhibits serotonin and noradrenaline uptake in rat synaptosomes to a similar degree (1.3 $\mu$M *vs* 5.8 $\mu$M respectively) (Samanin *et al.*, 1979) establishes that this ligand interacts with the noradrenaline transporter.

Radioiodinated derivatives of phenylpiperazines have been studied by several investigators. Hanson *et al.* (1985) have described the biodistribution of several radioiodinated phenylepiperazines including derivatives of 4-phenylpiperazine, the ganglionic-stimulating drug, 1,1-dimethyl-4-phenylpiperazinium (DMPP) (Hanson, 1982; Hanson *et al.*, 1983), 1-carboxamidino-4-([$^{125}$I]iodophenyl)piperazine (Hanson *et al.*, 1986) and 1-[$^{11}$C]methyl-4-aryl-piperazinium salts (Elmaleh *et al.*, 1993). These studies and others (Chumpradit *et al.*, 1989; Hanson and Hasan, 1987) have demonstrated that phenylpiperazine derivatives accumulate in a variety of tissues (brain, adrenal medulla and myocardium) to varying degrees.

These findings led to our investigation of the ability of these ligands to compete for MIBG uptake in cultured neuroblastoma. Initial results indicating that 1-carboxamidino-4-phenylpiperazine (CAPP) inhibits MIBG uptake by SK-N-BE(2C) cells prompted further investigations of this class of compounds as second generation or alternative radiopharmaceuticals for targeting neuroblastoma.

Correspondence: JW Babich, Division of Nuclear Medicine, Department of Radiology, Tilton-2, Massachusetts General Hospital, 32 Fruit Street, Boston MA 02114, USA
Received 27 July 1995; revised 11 April 1996; accepted 17 April 1996

The aims of this study were: (1) to evaluate the ability of selected phenylpiperazine derivatives to compete with MIBG uptake by cultured neuroblastoma cells; (2) to evaluate the uptake and retention of a model phenylpiperazine, namely, 4-(3-[$^{125}$I]iodophenyl) piperazine, in cultured neuroblastoma cells; and (3) to compare the biodistribution of 4-(3-[$^{125}$I]iodophenyl) piperazine with [$^{125}$I]MIBG in rats.

### Materials and methods

### *Materials*

All reagents were obtained as the highest available grade from commercial sources. 4-(3-chlorophenyl)-piperazine (mCPP), 4-phenylpiperazine (PP), 4-(3-trifluoro methyl phenyl)-piperazine (TFMPP) (1,1-dimethyl-4-phenyl)-piperazinium hydrochloride (DMPP) and the piperidine derivative chlorophenyl hydroxypiperidine (CP(OH)P), desmethylimipramine (DMI) and serotonin (5HT) were obtained from Sigma Chemical Co. 1-Carboxamidino-4-phenylpiperazine (CAPP) was prepared according to previously described methods (Hanson, 1982). Structures of the ligands used in this study are shown in Figure 1.

### *Synthesis of 1-carboxamidino-4-phenyl-piperazine (CAPP)*

4-Phenyl-piperazine hydrochloride (2.00 g), triethylamine (2.50 g) and methylthiopseudourea sulphate (1.50 g) were added to a round bottom flask and the solution was refluxed at 100°C for 6 h using an oil bath. The reaction mixture was then cooled to room temperature and transferred to an ice bath. On cooling the product crystallised. The precipitate was filtered, washed twice with ice-cold water, transferred to a clean preweighed round bottom flask and dried under vacuum overnight. The yield of dry product was 0.54 g. The product was dissolved in 30 ml of 2N HCl and excess HCl was removed by rotary evaporation. The resulting salt was dried under vacuum overnight. The yield of final product was 0.42 g (~17%). The product was characterised by silica gel thin layer chromatography developed with ethanol–ethyl acetate–ammonium hydroxide, 20:20:1, melting point and $^{1}$H NMR [Varian model 300XL (300 MHz)].

### *Synthesis and radiolabelling of MIBG*

MIBG was prepared according to the methods of Wieland *et al.* (1980). [$^{125}$I]MIBG was prepared by a modification of the procedure reported by Mangner *et al.* (1982). Radiochemical purity was determined before experimental use. Only preparations with purities of greater than 95% were used. Typically, the specific activity of the radiopharmaceutical was approximately 5 mCi mg$^{-1}$.

### *Radioiodination of 4-(3-chloro-phenyl)-piperazine (mCPP)*

[$^{125}$I] labelled 4-(3-iodo-phenyl)-piperazine(IPP) was prepared using the solid phase halogen exchange reaction described by Manger *et al.* (1982) and later applied to iodo- for chloro-substitution (Gildersleeve, *et al.*, 1989). One milligram of mCPP in 0.3 ml of water was added to a sterile glass vial followed by 0.3 ml of a 0.1 M ammonium sulphate solution. To this mixture was added [$^{125}$I]NaI (Amersham International) in a volume of 20–50 μl of dilute sodium hydroxide. The vial was sealed with a Teflon/silicone septa and aluminium crimp top, vented with a 20-gauge butterfly needle attached to a 20 ml syringe and placed in a dry heating block at 175°C for 1 h. The dry mixture was initially reconstituted with 1 ml of distilled water and the efficiency of radioiodine incorporation was assayed by TLC using TLC-SG (Merck) as stationary phase and ethanol–ethyl acetate–ammonium hydroxide (20:20:1) as mobile phase. In this system IPP is retained at the origin ($R_f = 0.0$) and $I^-$ migrates with $R_f = 0.8$. Radioiodine incorporation was approximately 78%. Removal of unreacted radioiodine was accomplished by anion exchange chromatography (Ott *et al.*, 1992). Briefly, the reaction product was dissolved in 0.5 M acetate buffer (pH 5) and passed through a sterile anion exchange membrane (AG1-X8, BioRad) in the carbonate form. The final product was passed through a Millex-GS 0.22 μm filter (Millipore Corp.). The radiochemical purity of the final product was >95%.

### *Cell culture*

The human neuroblastoma cell line SK-N-SH was obtained from the American Type Culture Collection (ATCC,

**Figure 1** Structures of phenyl piperazine ligands used in this study.

**Figure 2** The effect of increasing concentration of phenylpiperazine ligands on the uptake of MIBG by SK-N-BE(2C) cells. The order of affinity for inhibition of MIBG uptake was IPP$\geqslant$CAPP=CPP>DMPP>CP(OH)P>TFMPP. The standard error of the mean for each data point was $\leqslant$10%.

Bethesda, MD, USA). The human neuroblastoma cell line SK-N-BE(2C) was kindly provided as a gift from Dr J Beidler. The cell lines were grown in T125 Nunc flasks containing Eagle's medium, 10% fetal calf serum (FCS) and 20 mM Hepes buffer (pH 7.3). These cell lines have previously been shown to be of neuroendothelial origin and to have characteristic neuroblastoma-like morphology and histochemistry (Biedler *et al.*, 1990). Bulk quantities of cells were grown in Falcon tissue culture flasks (75 $cm^2$) in a 5% carbon dioxide/air atmosphere in a humidified water jacketed incubator at 37°C. The culture medium was minimal essential medium (MEM) supplemented with glutamine, Earl's salts, sodium pyruvate, penicillin, streptomycin, Hepes (10 mM) and fetal bovine serum (10%), pH 7.4.

For the experiments described below, cells were harvested using trypsine/versine (Sigma Chemical Co.) and plated into multiwell tissue culture plates (24 deep well, flat bottom, tissue culture-treated polystyrene plates, Falcon) at a concentration of approximately $5 \times 10^5$ cells per well. The cells were grown to near confluence in the medium described above. In most cases, experiments were performed 24 h after plating. Whenever possible experiments were performed with both cell lines.

Ten minutes before use of the cells, the medium was changed to Eagle's salt solution containing 20 mM Hepes without FCS (Eagle's/Hepes). The medium was heated to 37°C before use. Ligand and [$^{125}$I]MIBG were allowed to incubate in the presence of the cells as described below. After the appropriate incubation period the medium was removed and the cells were then rinsed with 1 ml of ice-cold Eagle's/Hepes to remove non-specifically bound MIBG. The cells were harvested using trypsine/versine. Initial incubation medium, wash and cell pellet were counted for radioactivity using a well-type gamma counter (MR80 Kontron, UK or LKB model no. 1282, Wallac Oy, Finland) set to the [$^{125}$I] gamma energy. Cell number was determined with a haemocytometer. All experiments were performed in triplicate.

### *Competitive inhibition of MIBG uptake by phenylpiperazines*

The ability of selected phenylpiperazines to inhibit MIBG uptake by the neuroblastoma cell line SK-N-BE(2C) was determined by incubating the cells with [$^{125}$I]MIBG (0.1 $\mu$M) for 2 h in the presence of varying concentrations of test ligand. In previous studies it has been shown that MIBG uptake apparently reaches 90% of maximum within 2 h. Determination of the percentage binding of MIBG to cells in the presence of competing ligand was determined by expressing cell-associated MIBG radioactivity as a percentage of total radioactivity. These values were compared with the percentage MIBG uptake in the absence of competing ligand (control). All values were normalised to the average number of cells. The concentration of competing ligand which inhibited MIBG binding by 50% of the control value ($IC_{50}$) was determined by graphical analysis.

### *Uptake and retention of [$^{125}$I]IPP by neuroblastoma cells*

The potential of phenylpiperazines as targeting agents for neuroblastoma is suggested by the ability of these compounds to inhibit the uptake of [$^{125}$I]MIBG *in vitro*. In order to understand better the potential of these compounds for targeting neuroblastoma it was necessary to characterise the uptake and retention behaviour of a model phenylpiperazine. 4-(3-[$^{125}$I]iodophenyl)-piperazine was chosen for evaluation on the basis of the *in vitro* competition studies (see below) which indicated that 4-(phenyl)-piperazine and the chloro analogue, CPP, were more effective than CAPP and other phenylpiperazines in inhibiting MIBG uptake.

The uptake of [$^{125}$I]IPP by SK-N-SH and SK-N-BE(2C) cells was studied as a function of time. For these studies, cells were incubated in the presence of [$^{125}$I]IPP (0.1 $\mu$M) and uptake was determined at various times. Retention was studied by incubating the cells in the presence of [$^{125}$I]IPP (0.1 $\mu$M) for 2 h, followed by removal of the [$^{125}$I]IPP containing medium and replacement with fresh medium (no [$^{125}$I]IPP) and determination of cell bound radioactivity at various times.

Studies of the selectivity of [$^{125}$I]IPP uptake and characterisation of the transport system involved were also performed. For these studies, cells were co-incubated in the presence of 0.1 $\mu$M [$^{125}$I]IPP and one of the following compounds: MIBG, DMI, 5HT, TFMPP, CPP and IPP in order to determine the ability of these agents to inhibit [$^{125}$I]IPP uptake. The phenylpiperazines with greatest affinity for the transporter should be preferred ligands for further development. The degree of inhibition by MIBG should indicate if the transporter is similar to that responsible for MIBG uptake. Since phenylpiperazines are known to interact with serotonin (5HT) receptor sites, 5HT was studied as a competitive inhibitor of [$^{125}$I]IPP uptake. The well-known Uptake-1 inhibitor, desmethylimipramine (DMI), was also studied.

### *Biodistributions of [$^{125}$I]IPP and [$^{125}$I]MIBG in normal rats*

The biodistributions of [$^{125}$I]4-(3-iodophenyl)-piperazine ([$^{125}$I]IPP) and [$^{125}$I]MIBG were measured in separate groups of animals. Determination of the distribution of [$^{125}$I]IPP in normal animals will give an indication of expected target to normal tissue ratios compared with MIBG. This data, in conjunction with the results of the *in vitro* experiments, should establish the feasibility of using phenylpiperazines for imaging neuroblastoma.

Biodistribution studies were performed in groups of thirty normal male Sprague–Dawley rats weighing approximately 150 g (Charles River Breeding Laboratories, Burlington, MA, USA). The animals were injected intravenously (via tail vein) with approximately 5 $\mu$Ci (185 kBq) of each tracer to determine biodistribution at 5, 30, 60, 120 and 1440 min (each compound was evaluated in six animals at each time point). Samples of blood, heart, lung, liver, spleen, kidney, adrenal, stomach, gastrointestinal tract, testes, skeletal muscle, bone and brain were weighed and radioactivity was measured with a well-type gamma counter (LKB model no. 1282, Wallac Oy, Finland). To correct for radioactive decay, aliquots of the injected doses were counted simultaneously. The results were expressed as percentage injected dose per gram and percentage injected dose per organ as well as target to background ratios (defined as percentage injected dose per gram adrenal/percentage injected dose per gram normal tissue).

### *Statistical methods*

The results of the biodistribution studies were evaluated by analyses of variance (ANOVA) with a linear model in which compound and time were the classification variables: %ID $g^{-1}$ or %ID per organ = Compound + Time + Compound*Time. *Post hoc* comparisons were performed by Duncan's new multiple range test (Duncan 1955). The first subscript of each F value is the number of degrees of freedom for: the first classification variable $(n-1)$, the second classification variable $(m-1)$ or the interaction $[(n-1) \times (m-1)]$. The second subscript is the number of residual degrees of freedom (total number of observations, $n \times m$). All results are expressed as mean ± the standard error of the mean (s.e.m.).

### Results

### *Synthesis of CAPP*

CAPP was prepared by the method of Hanson (1982). Silica gel thin-layer chromatography (TLC-sg) showed the presence of a single UV absorbing band with an $R_f$ of 0.05. Proton NMR was in agreement with literature values.

### *Radiolabelling of iodophenyl piperazine ([$^{125}$I]IPP)*

Radioiodine incorporation into chlorophenyl-piperazine was >75% as determined by TLC-sg using ethanol–ethyl acetate–20% ammonium hydroxide (20:20:1) as mobile phase. Removal of unreacted radioiodine was accomplished by passing the reaction mixture through an anion exchange resin membrane (BioRad AG1-X8). After purification, radiochemical purity was always greater than 95% as determined by radio-TLC. In this system the $R_f$ of radioiodinated phenylpiperazine was 0.05, and $I^-$ migrates with $R_f = 0.8$.

### *Radiolabelling of Metaiodobenzylguanidine (MIBG)*

The radiochemical purity of [$^{125}$I]MIBG was always greater than 95% as determined by TLC-sg (Merck) as stationary phase and ethanol–ethyl acetate–ammonium hydroxide (20:20:1) as mobile phase. In this system MIBG stays at the origin ($R_f = 0.05$) and $I^-$ migrates with $R_f = 0.8$.

### *Competitive binding with [$^{125}$I]MIBG*

Figure 2 shows the effect of increasing concentrations of phenylpiperazine ligands on the uptake of MIBG into SK-N-BE(2C) cells. The concentrations of each ligand required to produce a 50% inhibition of MIBG uptake are summarised in Table I. IPP, CAPP and mCPP had similar $IC_{50}$ values, within an order of magnitude of MIBG itself. DMPP was slightly less potent than CAPP and mCPP. CP(OH)P and TFMPP had potencies which were approximately 100- and 200-fold lower than the other phenylpiperazines studied. Hence, the order of potency for inhibition of MIBG uptake was PP ⩾ CPP = CAPP > DMPP > CP(OH)P > TFMPP ($P < 0.05$).

### *In vitro evaluation of [$^{125}$I]3-iodophenylpiperazine in neuroblastoma*

The uptake of [$^{125}$I]IPP into the neuroblastoma cell lines, SK-N-BE(2C) and SK-N-SH, was studied as a function of time using a fixed concentration of IPP (0.1 μM). The uptake kinetics of [$^{125}$I]IPP are shown in Figure 3. The rate of uptake was similar in both cell lines and was greatest over the time period 0 to 60 min. After 60 min the rate decreased.

The retention of [$^{125}$I]IPP by SK-N-SH and SK-N-BE(2C) cells was studied after a 2 h incubation followed by removal of the medium containing [$^{125}$I]IPP and replacement with fresh medium without [$^{125}$I]IPP. After an early rapid washout of ~50% of the initial radioactivity, retention remained constant up to 3 h (data not shown). The uptake and retention of [$^{125}$I]IPP is similar to the behaviour of MIBG over the same time interval (Babich, 1994).

The ability of the halogenated phenylpiperazines to inhibit [$^{125}$I]IPP uptake into both neuroblastoma cell lines was evaluated with increasing concentrations of ligand. IPP self-inhibited [$^{125}$I]IPP at approximately 10 fold lower concentration than MIBG in both cell lines. These data indicate that IPP has a relatively high affinity for these cell lines with approximate $IC_{50}$s of 3 μM, a concentration similar to that required for inhibition of [$^{125}$I]MIBG (2.5 μM) uptake. The significance of the higher concentration of MIBG required to inhibit [$^{125}$I]IPP uptake is unclear since MIBG self-inhibits at a concentration of approximately 0.4 μM. However, the observed difference may be due to more rapid association of IPP with the transporter rather than a thermodynamic effect. A prolonged incubation period may clarify these differences in apparent affinity.

Serotonin produced only a slight reduction in uptake at the highest concentrations studied. Although not statistically significant, this effect appeared to be slightly more pronounced in SK-N-BE(2C) cells. In contrast, DMI was a potent inhibitor of [$^{125}$I]IPP uptake in both cell lines. This strongly suggests that [$^{125}$I]IPP uptake is mediated via the uptake-1 transporter. The $IC_{50}$s of DMI were 1.5 μM and 0.6 μM for SK-N-BE(2C) and SK-N-SH respectively. The $IC_{50}$s of 5HT were >100 μM in both cell lines.

In order to characterise the structural preferences of the transporter which is responsible for [$^{125}$I]IPP uptake, the abilities of TFMPP, CPP and IPP to inhibit [$^{125}$I]IPP uptake

**Table I** $IC_{50}$s for competitive inhibtion of [$^{125}$I]MBG uptake by neuroblastoma cell line SK-N-BE(2C) of phenylpiperazines

| *Compound* | *$IC_{50}$ (μM)* |
|---|---|
| DMPP | 5 |
| PP | 1.5 |
| CPP | 2.5 |
| CAPP | 2.5 |
| CP(OH)P | 30 |
| TFMPP | 65 |
| MIBG | 0.3 |

**Figure 3** Uptake of [$^{125}$I]3-iodophenylpiperazine by neuroblastomas cells in culture. Cells ($10^6$ per well) were incubated in the presence of 0.1 μM [$^{125}$I]IPP for various time intervals. Error bars smaller than the symbols are not shown.

were studied. In both cell lines, IPP was the most potent inhibitor of [$^{125}$I]IPP uptake, followed by mCPP and TFMPP. This finding supports the choice of [$^{125}$I]IPP as an initial radiolabelled tracer for the study of this class of compounds and reflects the previous finding that TFMPP has a significantly lower ability to inhibit MIBG uptake compared with mCPP or IPP. The $IC_{50}$s for IPP, CPP and TFMPP were 2.5, 7.0 and >20 $\mu$M for SK-N-BE(2C) and 1.8, 9.0 and 40 $\mu$M for SK-N-SH respectively. The $IC_{50}$s for MIBG were 18 and 25 $\mu$M for SK-N-BE(2C) and SK-N-SH respectively.

**Table II** Biodistribution of [$^{125}$I]IPP and [$^{125}$I]MIBG in the rat

| *Organ* | *Time (min)* | *IPP* | *MIBG* |
|---|---|---|---|
| Blood | 5 | 0.16±0.012 | 0.13±0.010 |
| | 30 | 0.28±0.004 | 0.10±0.002 |
| | 60 | 0.30±0.026 | 0.12±0.000 |
| | 120 | 0.25±0.013 | 0.10±0.000 |
| | 1440 | 0.03±0.002 | 0.02±0.001 |
| Heart | 5 | 1.31±0.058 | 2.96±0.237 |
| | 30 | 0.46±0.017 | 1.94±0.082 |
| | 60 | 0.44±0.041 | 1.68±0.109 |
| | 120 | 0.21±0.006 | 1.18±0.066 |
| | 1440 | 0.03±0.002 | 0.09±0.004 |
| Lung | 5 | 9.41±0.355 | 3.79±0.196 |
| | 30 | 3.38±0.246 | 2.08±0.078 |
| | 60 | 2.45±0.134 | 1.66±0.122 |
| | 120 | 0.79±0.037 | 0.95±0.029 |
| | 1440 | 0.05±0.003 | 0.09±0.007 |
| Liver | 5 | 1.13±0.072 | 0.95±0.073 |
| | 30 | 1.52±0.069 | 0.70±0.032 |
| | 60 | 1.56±0.059 | 0.43±0.021 |
| | 120 | 1.14±0.029 | 0.34±0.016 |
| | 1440 | 0.33±0.007 | 0.04±0.002 |
| Spleen | 5 | 0.10±0.096 | 0.89±0.084 |
| | 30 | 0.75±0.026 | 0.70±0.032 |
| | 60 | 0.60±0.031 | 0.58±0.041 |
| | 120 | 0.25±0.010 | 0.61±0.037 |
| | 1440 | 0.03±0.001 | 0.07±0.006 |
| Kidney | 5 | 1.25±0.057 | 1.10±0.140 |
| | 30 | 0.94±0.036 | 0.48±0.019 |
| | 60 | 1.11±0.055 | 0.41±0.025 |
| | 120 | 0.74±0.027 | 0.35±0.027 |
| | 1440 | 0.11±0.009 | 0.05±0.003 |
| Adrenal | 5 | 1.58±0.081 | 1.30±0.168 |
| | 30 | 1.01±0.077 | 1.17±0.039 |
| | 60 | 1.21±0.071 | 1.03±0.029 |
| | 120 | 0.40±0.025 | 1.33±0.092 |
| | 1440 | 0.08±0.005 | 0.79±0.070 |
| Stomach | 5 | 0.45±0.027 | 0.31±0.029 |
| | 30 | 0.10±0.059 | 0.23±0.030 |
| | 60 | 1.71±0.101 | 0.60±0.035 |
| | 120 | 2.11±0.126 | 0.68±0.087 |
| | 1440 | 0.15±0.012 | 0.19±0.021 |
| GI tract | 5 | 0.41±0.019 | 0.36±0.078 |
| | 30 | 0.62±0.035 | 0.59±0.020 |
| | 60 | 0.65±0.044 | 0.58±0.030 |
| | 120 | 0.75±0.074 | 0.61±0.024 |
| | 1440 | 0.05±0.004 | 0.09±0.006 |
| Testes | 5 | 0.24±0.015 | 0.13±0.006 |
| | 30 | 0.38±0.016 | 0.18±0.006 |
| | 60 | 0.36±0.007 | 0.14±0.004 |
| | 120 | 0.29±0.008 | 0.15±0.009 |
| | 1440 | 0.01±0.001 | 0.03±0.003 |
| Muscle | 5 | 0.44±0.019 | 0.28±0.027 |
| | 30 | 0.23±0.014 | 0.34±0.022 |
| | 60 | 0.18±0.014 | 0.33±0.022 |
| | 120 | 0.12±0.005 | 0.36±0.026 |
| | 1440 | 0.01±0.005 | 0.04±0.004 |
| Bone | 5 | 0.38±0.021 | 0.32±0.015 |
| | 30 | 0.30±0.015 | 0.34±0.021 |
| | 60 | 0.26±0.013 | 0.24±0.010 |
| | 120 | 0.15±0.020 | 0.27±0.020 |
| | 1440 | 0.01±0.009 | 0.02±0.003 |
| Brain | 5 | 0.84±0.035 | 0.05±0.005 |
| | 30 | 0.85±0.051 | 0.03±0.002 |
| | 60 | 0.68±0.036 | 0.03±0.002 |
| | 120 | 0.32±0.010 | 0.02±0.002 |
| | 1440 | 0.01±0.001 | 0.004±0.000 |

Percentage injected doses per g (mean±s.e.m.).

*Biodistributions of [$^{125}$I]IPP and [$^{125}$I]MIBG in normal rats*

Based on the *in vitro* results, the biodistributions of [$^{125}$I]IPP and [$^{125}$I]MIBG were compared in normal rats. The results of these studies are summarised in Table II. With the exceptions of kidney, stomach and the gastrointestinal tract, the tissue concentrations of both compounds decreased with time.

In blood, ANOVA demonstrated significant main effects of compound ($F_{1,56}=83.48$, $P<0.0001$) and time ($F_{4,56}=29.23$, $P<0.0001$). However, compound by time interactions were not significant. The order of concentrations of the compounds in blood was IPP>MIBG. In cardiac tissue, ANOVA showed significant main effects of compound ($F_{1,52}=143.15$, $P<0.0001$), and time ($F_{4,52}=46.21$, $P<0.0001$), and compound by time interaction ($F_{9,64}=3.69$, $P<0.001$). The order of concentrations of the compounds was MIBG>IPP. In lung, ANOVA demonstrated significant main effects of compound ($F_{1,50}=21.29$, $P<0.0001$) and time ($F_{4,50}=45.50$, $P<0.0001$), and compound by time interaction ($F_{9,65}=13.80$, $P<0.0001$). The order of concentrations of the compounds was IPP>MIBG ($P<0.01$). In liver, ANOVA showed significant main effects of compound ($F_{1,53}=137.46$, $P<0.0001$) and time ($F_{4,53}=37.88$, $P<0.0001$), however compound by time interaction was not significant. As in lung, the order of concentrations of the compounds was IPP>MIBG. In spleen, ANOVA demonstrated a significant main effect of time ($F_{5,42}=71.87$, $P<0.0001$). In this organ, the main effect of compound was not significant ($F_{1,52}=2.41$). In kidney, ANOVA showed significant main effects of compound ($F_{1,54}=61.99$, $P<0.0001$) and time ($F_{4,54}=60.63$, $P<0.0001$), and compound by time interaction ($F_{9,69}=10.94$, $P<0.0001$). The order of concentrations of the compounds was IPP>MIBG. In adrenal, ANOVA demonstrated significant main effects of compound ($F_{1,49}=12.45$, $P<0.001$) and time ($F_{4,49}=16.54$, $P<0.0001$), and compound by time interaction ($F_{9,53}=5.56$, $P<0.0001$). The order of concentrations of the compounds was MIBG>IPP. In stomach, ANOVA demonstrated significant main effects of

**Figure 4** Uptake of [$^{125}$I]IPP and [$^{125}$I]MIBG by the adrenal gland of normal rats. These data demonstrate the similar levels of early uptake for both tracers with greater washout of IPP at later times. Error bars represent 1 standard deviation. Error bars smaller than the symbols are not shown.

compound ($F_{1,45}=51.75$, $P<0.0001$) and time ($F_{4,45}=24.90$, $P<0.0001$), and compound by time interaction ($F_{9,53}=5.56$, $P<0.0001$). The order of concentrations of the compounds was IPP>MIBG. In the gastrointestinal tract, ANOVA demonstrated significant main effects of time ($F_{4,52}=62.88$, $P<0.0001$), but showed no significant effect of compound ($F_{1,52}=3.927$). In testis, ANOVA demonstrated significant main effects of compound ($F_{1,50}=94.75$, $P<0.0001$) and time ($F_{4,50}=49.58$, $P<0.0001$), and compound by time interaction ($F_{9,67}=4.77$, $P<0.001$). The order of concentrations of the compounds was IPP>MIBG. In skeletal muscle, ANOVA showed significant main effects of compounds ($F_{1,47}=10.38$, $P<0.01$) and time ($F_{4,47}=22.97$, $P<0.0001$), however compound by time interaction was not significant. The order of concentrations of the compounds was MIBG>IPP. In bone, ANOVA demonstrated a significant main effect of time ($F_{4,48}=80.42$, $P<0.0001$) but demonstrated no significant main effect of compound ($F_{1,48}=0.002$). In brain, ANOVA demonstrated significant main effects of compound ($F_{1,51}=123.56$, $P<0.0001$), and time ($F_{4,51}=12.92$, $P<0.0001$) and compound by time interaction ($F_{3,68}=5.15$, $P<0.0001$). The order of concentrations of the compounds was IPP>MIBG.

At the early time points, uptake of both compounds was most prominent in the lung, heart and adrenals. Although maximal adrenal uptake of IPP was greater or equal to that of MIBG from 5 to 60 min after injection, by 2 h the concentration of [$^{125}$I]3-iodophenyl-piperazine dropped to

**Figure 5** Selected target to background ratios for IPP (–□–) and MIBG (....◇....) (defined as % injected dose per gram adrenal/% injected dose per gram other tissue).

approximately 0.4% per g, while MIBG remained at approximately 1.3% per g (Figure 4). The differences increased even further in favour of MIBG by 24 h after injection. The target–background (T/B) ratios for IPP increased with time in lung, spleen and heart. In blood and kidney the T/B ratios decreased with time. In contrast, T/B ratios for MIBG increased with time in most tissues (Figure 5).

### Discussion

In the present study we have demonstrated that it is possible to use an inhibition assay to screen for compounds which display affinity for neuroblastoma in a manner mechanistically similar to MIBG. Our results demonstrate that selected phenylpiperazines demonstrated good inhibition of [$^{125}$I]MIBG uptake by neuroblastoma cells. Of the phenylpiperazines studied, [$^{125}$I]IPP was also shown to accumulate in neuroblastoma. Our data support the concept that [$^{125}$I]IPP is taken up by a specific transporter which is saturable and that this uptake is strongly inhibited by the known uptake-1 inhibitor, DMI.

As phenylpiperazines are known to interact with 5-HT receptors, competition of [$^{125}$I]IPP with 5HT was also used to characterise uptake. The weak inhibition of uptake by 5HT indicates that the transporter responsible for IPP uptake is more specific for IPP than 5HT. Further work is needed to characterise IPP and its mode of uptake fully, as it has been reported that certain serotonin receptors are present on some neuroblastoma cell lines (Hoyer *et al.*, 1988; Watling, 1988), but not serotonin uptake transporters (Pranzatelli and Balletti, 1992). The strong inhibition of IPP uptake by DMI suggests such receptors may play only a minor role, if any.

As described above, at early times after injection, the distribution of IPP is not only similar to MIBG but of the same magnitude in tissues such as the heart, lung and adrenals. Although adrenal uptake of [$^{125}$I]IPP initially exceeded that of MIBG, its relatively rapid washout suggests that retention mechanisms may dominate at late time points. Differences in retention may be due to the effective charges associated with the secondary amine of IPP and the guanidino group of MIBG. Also there may be stronger binding of the guanidine moiety to intracellular targets such as carboxylate groups of oxoanions (Muller *et al.*, 1988; Wigley *et al.*, 1987).

The lack of stomach radioactivity of [$^{125}$I]IPP at later time points indicates that radiolabelling at the meta position, via iodine for chlorine exchange, provides a more stable radiopharmaceutical than direct iodination of phenylpiperazines in which the amino group at the 4 position of the piperazine ring directs labelling to the para position of the phenyl ring (Hanson *et al.*, 1986; Letiec *et al.*, 1986).

A potential drawback of IPP may be its significant uptake in the brain as well as its accumulation in the lung. Both organs would be particularly sensitive to high levels of radiation in the case of therapy with this agent. The accumulation of IPP in these organs may be reduced, however, by derivatising the amino group at the 1 position (as per CAPP). Changing this secondary amine to a quarternary amine would hamper its ability to cross the blood–brain barrier as would conversion to a carboxamidino group as demonstrated by the biodistribution data reported by Hanson for the radioiodinated compound (Hanson, 1982, 1985). A combination of iodination at the 3 position of the phenyl ring and alteration of the charge or basicity of the amino group at the 1 position of the piperazine ring, should provide a radiopharmaceutical with improved radiochemical stability and lower brain and lung uptake as well as similar *in vivo* characteristics to MIBG. In addition, the phenylpiperazine compounds may allow for further structural alterations and possible improvement over MIBG.

Recently, the feasibility of producing an alpha-emitting analogue of MIBG, [$^{211}$At]meta-astatinatobenzyl guanidine has been demonstrated (Vaidyanathan and Zalutsky, 1992). The rationale for developing this agent is the greater lethality of the alpha particle of $^{211}$At as compared with beta emissions of $^{131}$I. Although this compound is a good first step towards an agent with greater radiotherapeutic effectiveness, accumulation in normal organs will lead to high radiation burden in normal tissues and will probably limit its application. This is particularly true for the heart where impaired function could result in significant morbidity. In the present study we demonstrated that there is significant uptake of [$^{125}$I]IPP by neuroblastoma *in vitro* and decreased myocardial accumulation compared with MIBG *in vivo*. As a carrier for $^{211}$At, phenylpiperazines may offer reduced radiation burden to certain normal tissues as compared with MIBG.

In summary, the potential of a new class of compounds for targeting neuroblastoma has been demonstrated using competitive inhibition studies. This approach appears to be a reliable screening method for affinity for neuroblastoma cells via uptake-1. Phenylpiperazines have been shown to represent a class of compounds capable of inhibiting MIBG uptake into neuroblastoma cells at the $\mu$M level. We demonstrate here for the first time that specific radioiodinated phenylpiperazine analogues display significant uptake into neuroblastoma cells which appears to be saturable and inhibited by agents known to inhibit uptake-1. The biological distribution of [$^{125}$I]IPP is significantly different from MIBG in a variety of tissues and may represent a new avenue for exploitation in terms of therapy and diagnosis.

### References

ABUZAR S AND SHARMA S. (1984). Synthesis of 2-carbalkoxyamino-5(6)-(1-substituted piperazine-4-yl/piperazin-4-ylcarbonyl) benzimidazoles and related compounds as potential antihelmintics. *Pharmazie*, **39**, 747–749.

BABICH JW. (1994). A study of the uptake and retention mechanism of meta-iodobenzylguanidine in neuroblastoma cells. Ph.D. dissertation, University of London.

BAGDY G, SZEMEREDI K, KANYICSKA B AND MURPHY DL. (1989). Different serotonin receptors mediate blood pressure, heart rate, plasma catecholamine and prolactin responses to m-chlorophenylpiperazine in conscious rats. *J. Pharmacol. Exp. Therap.*, **250**, 72–78.

BIEDLER J, SPENGLER B AND ROSS R. (1990). Cellular maturation and oncogene expression during drug-induced differentiation *in vitro*: a brief review. *Eff. Therap. Biol. Kin. Res. Tum.*, **A**, 287–312.

CHUMPRADIT S, KUNG H, BILLINGS J, GUO Y, WU Y AND SHIH J. (1989). Preparation and biodistribution of 1-[2-(3[I-125] iodo-4-adminophenyl) ethyl]4-[3-(trifluoromethyl) phenyl) phenyl]piperazine and 1-[2-(3-[I-125] iodo-4-azidophenyl)ethyl]-4-[3-(trifluoromethyl) phenyl]piperazine. *J. Med. Chem.*, **32**, 543–547.

DUNCAN DB. (1955). Multiple range tests and multiple F-tests. *Biometrics*, **11**, 1–42.

ELMALEH DR, PADMANBHAN S, HASSAN MA, CORREIA JA, HERMAN LW, HANSON RN AND STRAUSS HW. (1993). Synthesis and evaluation of 1[C-11]methyl-4-aryl-piperazinium salts as myocardial imaging agents. *Nucl. Med. Biol.*, **20**, 427–433.

FRASER SE AND BONNER-FRASER M. (1991). Migrating neural crest cells in the trunk of the avian embryo are multipoint. *Development*, **112**, 913–920.

FULLER R. (1986). Substituted phenylpiperazines as serotonin agonists: structural determinants of potency and interaction with receptor subtypes. *Psychol. Bull.*, **22,** 825–829.

GILDERSLEEVE DL, LIN TY, WIELAND DM, CILIAX BJ, OLSON JM AND YOUNG AB. (1989). Synthesis of a high specific activity I-125-labeled analog of PK 11195, potential agent for SPECT imaging of the peripheral benzodiazepine binding site. *Int. J. Radiat. Appl. Instrument. B Nucl. Med. Biol.*, **16,** 423–429.

GLENNON RM, YOUSIF M, ISMAIEL A, EL-ASHMAWY M, HERNDON J, FISCHER J, SERVER A AND HOWIE K. (1991). Novel 1-phenylpiperazine and 4-phenylpiperidine derivatives as high-affinity sigma ligands. *J. Med. Chem.*, **34,** 3360–3365.

HANSON R. (1982). Radioiodinated 1-carboxamidino-4-phenylpiperazine: a potential adrenal and myocardial imaging radiopharmaceutical. *Int. J. Appl. Radiat. Isot.*, **33,** 629–632.

HANSON R. (1983). Preparation and evaluation of radioiodinated phenylpiperazines as adrenomedullary imaging agents. *Int. J. Nucl. Med. Biol.*, **10,** 219–222.

HANSON R. (1985). Radioiodinated 1-substituted-4-phenylpiperazines as potential brain imaging agents. *Int. J. Nucl. Med. Biol.*, **12,** 315–320.

HANSON R AND HASSAN M. (1987). Phenylpiperazine-based radiopharmaceuticals for brain imaging. Synthesis and evaluation of radioiodinated 1-Alkyl-4-phenylpiperazines, *J. Med. Chem.*, **30,** 29–34.

HANSON R, FRANKE L AND WEBB N. (1985). Radioiodinated 1-(diethylamino propyl)-4-phenylpiperazine: a potential brain imaging agent. *Int. J. Nucl. Med. Biol.*, **12,** 397–400.

HOEFNAGEL C, VOUTE P, DEKRAKER J AND MARCUSE H. (1985). Total-body scintigraphy with 131-meta-iodobenzylguanidine for detection of neuroblastoma. *Diagn. Imag. Clin. Med.*, **54,** 21–27.

HOYER D AND NEIJT HC. (1988). Identification of 5-HT3 recognition sites in membranes of N1E-115 neuroblastoma cells by radioligand binding. *Mol. Pharmacol.*, **33,** 303–309.

KENTROTI S, PRASAD KNI, CARVALHO E AND VERNADAKIS A. (1994). Differential regulation of phenotypic expression in a pluripotential neuroblastoma cell line. *Brain Res.*, **651,** 1–6.

KIMMIG B, BRANDEIS W, EISENHUT M, BUBECK B, HERMANN H AND WINKEL KZ. (1984). Scintigraphy of a neruoblastoma with 131 meta-iodobenzylguanidine. *J. Nucl. Med.*, **25,** 773–775.

LASHFORD LS, LEWIS IJ, FIELDING SL, FLOWER MA, MELLER S, KEMSHEAD JT AND ACKERY D. (1992). Phase I/II study of iodine 131 metaiodobenzylguanidine in chemoresistant neuroblastoma: a United Kingdom Children's Cancer Study Group investigation. *J. Clin. Oncol.*, **10,** 1889–1896.

LEITIEC A, GUILLOTEAU D, HUGUET F, BAULIEU J, BESNARD J AND VIEL C. (1986). Iodo carboxamidine-1 phenyl-4 piperazine un nouvel agent potentiel pour l'imagerie de la medullosurrenale: comparison avec le meta iodobenzylguadine. *Int. J. Nucl. Med. Biol.*, **12,** 495–496.

LLOYD K, DEPOORTERE H, SCATTON B, SHOEMAKER H, ZIVKOVIC B, MANOURY P, LANGER S, MORSELLI P AND BARTHOLINI G. (1985). Non-benzodiazepine anxiolytics: potential activity of phenylpiperazines without H-3-diazepam displacing action. *Pharmacol. Biochem. Behav.*, **23,** 645–652.

LYON R, TITELER M, MCKENNEY J, MAGEE P AND GLENNON R. (1986). Synthesis and evaluation of phenyl- and benzoylpiperazines as potential serotonergic agents. *J. Med. Chem.*, **29,** 630–634.

MCEWAN A, SHAPIRO B, SISSON J, BEIERWALTES W AND ACKERY D. (1985). Radio-iodobenzylguanidine for the scintigraphic location and therapy of adrenergic tumours. *Semin. Nucl. Med.*, **15,** 132–153.

MAJ J AND LEWANDOWSKA A. (1980). Central serotonin-mimetic action of phenylpiperazines. *Pol. J. Pharmacol. Pharm.*, **32,** 495–504.

MANAGER T, WU J AND WIELAND D. (1982). Solid-phase exchange radioiodination of aryl iodides. Facilitation by ammonium sulfate. *J. Org. Chem.*, **47,** 1484–1488.

MULLER G, RIEDE J AND SCHMIDTCHEN F. (1988). Host–guest bonding of oxoanions to guanidinium anchor groups. *Angew Chem.*, **11,** 1516–1519.

OEPEN G, BORK A, JAKOVLEV V, NICKEL B, THIEMER K AND ENGEL J. (1988). New analgesically-active N-acylated phenylpiperazines. *Arnzneimittle*, **38,** 1549–1552.

OTT RJ, TAIT D, FLOWER MA, BABICH JW AND LAMBRECHT RM. (1992). Treatment planning for 131I-MIBG radiotherapy of neural crest tumours using 124I-MIBG positron emission tomography. *Br. J. Radiol.*, **65,** 787–791.

PAWOWSKI L. (1983). A serotonergic component in the central action of 1-(o-methoxyphenyl)-piperazine. *Pol. J. Pharmacol. Pharm.*, **34,** 319–326.

PETTIBONE DJ AND WILLIAMS M. (1984). Serotonin-releasing effects of substituted phenylpiperazines *in vitro*. *Biochem. Pharmacol.*, **33,** 1531–1536.

PRANZATELLI MR AND BALLETTI J. (1992). Serotonin receptors in human neuroblastoma: a possible biologic tumor marker. *Exp. Neuro.*, **115,** 423–427.

SAMANIN R, MENNINI T, FERRARIS A, BENDOTTI C, BORSINI F AND GARATTINI S. (1979). Chlorophenylpiperazine: a central serotonin agonist causing powerful anorexia in rats. *Naunyn-Schmiedeberg's Arch. Pharmacol.*, **308,** 159–163.

TRONCONE L, RUFINI V, MONTEMAGGI P, DANZA F, LASORELLA A AND MASTRANGELO R. (1990). The diagnostic and therapeutic utility of radioiodinated metaiodobenzylguanidine (MIBG). *Eur. J. Nucl. Med.*, **16,** 325–335.

VAIDYANATHAN G AND ZALUTSKY MR. (1992). 1-(m-[211At]-astatobenzyl) guanidine: synthesis via astato demetallation and preliminary *in vitro* and *in vivo* evaluation. *Bioconj. Chem.*, **3,** 499–503.

WATLING KJ. (1988). Radioligand binding studies identify 5-HT3 recognition sites in neuroblastoma cell lines and mammalian CNS (review). *Trends Pharmacol. Sci.*, **9,** 227–229.

WIELAND D, WU J, BROWN L, MANGNER T, SWANSON D AND BEIERWALTES W. (1980). Radiolabelled adrenergic neuron-blocking agents: adrenomedullary imaging with I-131 iodobenzylguanidine. *J. Nucl. Med.*, **21,** 349–353.

WIGLEY D, LYALL A, HART K AND HOLBROOK J. (1987). The greater strength of arginine:carboxylate over lysine carboxylate ion pairs implications for the design of novel enzymes and drugs. *Biochem. Biophys. Res. Commun.*, **149,** 927–929.